# Barriers to Oral Health Care for Autistic Individuals—A Scoping Review †

**DOI:** 10.3390/healthcare12010103

**Published:** 2024-01-02

**Authors:** Jayne Jones, Elysa Roberts, Deborah Cockrell, Denise Higgins, Dileep Sharma

**Affiliations:** 1 Discipline of Oral Health, Oral Health School of Health Sciences, College of Health, Medicine and Wellbeing, University of Newcastle, Ourimbah, NSW 2258, Australia; jayne.jones@newcastle.edu.au (J.J.); deborah.cockrell@newcastle.edu.au (D.C.); denise.higgins@newcastle.edu.au (D.H.); 2 Discipline of Occupational Therapy, College of Health, Medicine and Wellbeing, University of Newcastle, Callaghan, NSW 2308, Australia; elysa.roberts@newcastle.edu.au

**Keywords:** autism spectrum disorder, parents, carers, dental practitioners

## Abstract

Background: Challenges in providing adequate dental care for individuals with Autism Spectrum Disorder (ASD) are recognised by parents, caregivers, and dental practitioners, leading to a higher prevalence of unaddressed dental needs. This scoping review aims to explore existing research on the obstacles to oral health care as perceived by individuals with ASD, as reported by their parents, caregivers, and dental professionals. Methods: Systematic searches were conducted in DOSS, Medline, and PubMed databases using relevant keywords to identify relevant studies. Barriers identified within these studies were then categorised based on themes identified. Results: The initial search yielded a total of 254 studies. Following the removal of duplicates and screening of titles and abstracts, 47 studies were further assessed against predetermined criteria, ultimately resulting in the inclusion of 16 articles in this scoping review. The identified barriers were grouped into five overarching themes: challenges in accessing appropriate care (n = 8), negative past experiences (n = 5), parental perceptions of the impact of ASD (n = 8), clinician bias (n = 2), and clinician education (n = 7). Conclusions: The findings of this review highlight the obstacles faced by individuals with ASD in obtaining routine oral health care. These results underscore the imperative for the development, testing, and implementation of tailored interventions focused on autism, as well as their integration into educational curricula for dental practitioners at various educational levels. This approach aims to enhance the delivery of equitable oral health care to individuals with ASD, starting from undergraduate through to postgraduate dental education.

## 1. Introduction

Autism spectrum disorder (ASD) is defined by the American Psychiatric Association in the Diagnostic and Statistical Manual of Mental Disorders (DSM5) as “persistent deficits in social communication and social interaction across multiple contexts, accompanied by restricted, repetitive patterns of behavior, interests, or activities” [1]. The prevalence of ASD cases varies worldwide due to inconsistency in diagnostic tools used, age ranges assessed and reporting requirements [2]. Some professionals prefer the term Autism Spectrum Condition (ASC) to move away from the negative image associated with Autism spectrum ‘disorder’ [3]. Diagnoses of ASD are classified into three levels based on the complexity of a person’s language, their social and support needs, and the presentation of their repetitive behaviors [1,4]. Significantly higher rates of individuals are being diagnosed with ASD worldwide including in Australia as noted in the Australian Bureau of Statistics report with 205,200 Australians (2018) diagnosed with ASD, a 25.1% increase from 164,000 individuals previously (2015) [5].

Recently, the Australian Senate established an inquiry into the services, support and life outcomes for Autistic Australians and a Select Committee on Autism was set up [6]. This committee supported the development of a National Autism Strategy for improving the health services for autistic people by targeting action to support the challenges and disadvantages that this population of people are facing [6]. A recent report identified that over 50% of Autistic individuals did not access the assistance they needed, resulting in unmet needs [5]. The report showed that Autistic people need assistance with at least one activity a day and that this includes daily assistance with cognitive and emotional tasks (39.2%), self-care (30.9%) and mobility (33.2%) [5]. Whilst requiring assistance with cognitive and emotional tasks, self-care, and mobility for autistic children would be consistent with the need for neurotypical children this need for assistance relates to the total Australian population of those individuals with a diagnosis of ASD. In 2018 the highest prevalence of ASD was in the 5 to 19 age groups (the prevalence was reported as 3.1% for 5–9 years, 3.3% for 10–14 years and 2.8% for 15–19 years [5]). The autism prevalence rates in Australia are higher amongst children and younger people which is influenced by age-related factors which affect the identification of the diagnosis of ASD [5].

One aspect of self-care that can be significantly impacted by ASD is oral health. Among various proposed reasons for oral health issues, are factors related to the restrictive repetitive patterns of behavior or atypical responses to sensory input associated with ASD that can increase the difficulty of plaque removal from the smooth and interproximal surfaces of teeth [7,8,9,10,11]. One aspect of atypical responses to sensory input for Autistic individuals is reduced intraoral sensory tolerance which makes it difficult to manage self-care or attend dental assessment and treatment appointments [7,8,9,10,11]. Parents, carers, and dental practitioners identified difficulties communicating and negotiating behavioral and sensory features of Autistic individuals as a contributor to a higher incidence of unmet dental needs [12,13,14].

It is also reported that Autistic individuals experience a higher prevalence of poor oral health due to periodontal disease and self-injuring behavior like trauma and bruxism [13]. In a systematic review by Silva et al., it was suggested that the incidence of periodontal disease in children and young adults with ASD is high with the prevalence of periodontal disease reported to be higher than 69% for Autistic individuals [15]. Interestingly, one study reported that the Oral Health Quality of Life (OHRQoL) of Autistic patients is higher than that of non-autistic children based on parents’ perceptions [16]. However, this finding appears to be due more to parents not prioritising oral health over other quality-of-life matters [16].

Negative perceptions of individuals with neurodevelopmental disabilities by dental professionals have been described as a barrier to oral health care for individuals with disabilities [17]. Hsu et al. called for a change in the attitude of oral health care providers and the development of further training in the form of continuing education of the oral health clinicians who are needed to treat these individuals [17]. An emerging body of evidence suggests a need for modifications to dental treatment for individuals with special needs, such as those associated with ASD [18,19]. Nelson et al. examined the impact of desensitisation on oral care for autistic children and recommended that at the first visit, the patient simply observed and experienced the treatment environment to allow them to “participate without anxiety brought on by the unknown” [20]. Adapting the clinical experience to the individual patient’s needs and developing familiarity with a consistent experience, promoted cooperation. Collectively, the ways to improve oral health for Autistic individuals are multifaceted and involve modifications by dental practitioners [20,21] and parents or carers [22], as well as service delivery and environmental modifications [7,11,23,24]. Further research in this space is needed to understand the barriers interfering with improved oral health and access to adequate dental care, and the need for a multidisciplinary approach to oral health care [25]. Hence, the purpose of this review was to evaluate and consolidate the current literature on barriers to oral health care for Autistic individuals as identified by parents, carers, and clinicians.

This scoping review was conducted to identify barriers rather than provide recommendations to adapt to the needs of autistic people in the dental environment. Its aim is to collate the literature that identifies the barriers perceived by parents and carers, autistic individuals and treating clinicians to enable health practitioners to adapt to the individual needs of all patients, including Autistic individuals who require a sensory-adapted experience and environment when attending for dental treatment.

## 2. Methods

This scoping review was performed based on the framework by Arksey and O’Malley for conducting the systematic search of the literature, classifying or charting the data and summarising and reporting the outcomes [26].

This review was conducted with the focus question: “what are the barriers to oral health care for Autistic individuals from the perspective of the individual, reported by parents, carers and dental practitioners”?

Studies for inclusion were sourced from DOSS (Dental and Oral Sciences), Medline and PubMed databases between 2014 and 2022. Articles published prior to 2014 were excluded because the DSM-5 was released in May 2013 and the diagnostic criteria for ASD were then redefined [1]. The database search was supplemented by hand searching. Relevant keywords used individually and in various combinations within the search strategy included Asperger, Autism Spectrum disorder, developmental disorder, oral health, oral hygiene, dental health, oral health care and oral care. Inclusion criteria were peer-reviewed, published papers that were written in English, available full text, and included any aspect on the topic of access and barriers to oral health care for Autistic individuals. Study designs included were case-controlled studies, cross-sectional studies, clinical studies, qualitative descriptive studies, and randomised controlled trials. Case reports, case series, reviews including systematic reviews, and opinion pieces were excluded from this review. Table 1 summarizes the inclusion and exclusion criteria.

**Figure 1:**
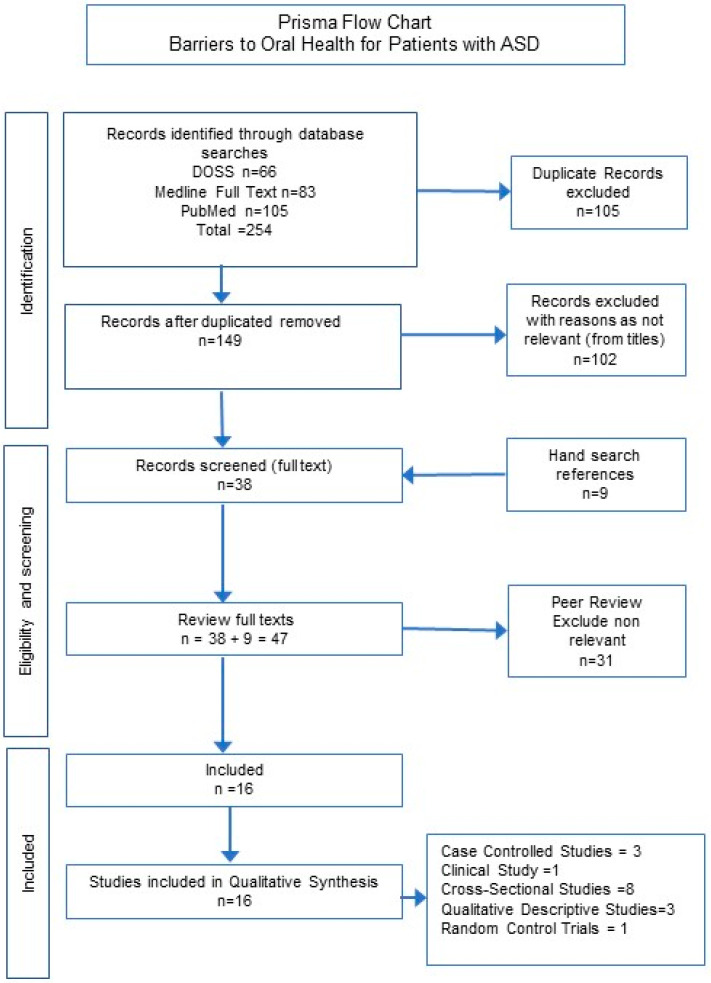
PRISMA flow chart illustrating process for data selection followed in this scoping review.

Data from each included study were extracted into a Microsoft Excel^TM^ spreadsheet. All papers were reviewed, and findings were categorised into thematic classifications aligned with the research question. The review team discussed and validated the themes and classification used across the included papers. The PRISMA flowchart summarizes the process followed in this review (Figure 1).

## 3. Results

A total of 254 studies were found through initial database searches including DOSS (n = 66), Medline (n = 83) and PubMed (n = 105). Subsequently, duplicates were removed, resulting in a total of 149 studies. Further 102 studies were excluded upon the title and abstract screening, resulting in 38 papers. A further nine papers were identified through hand searching of references. Finally, 47 full-text papers were reviewed and a further 31 were excluded as they did not fulfil the inclusion criteria resulting in 16 articles meeting the criteria for inclusion in this scoping review (Table 2).

The study characteristics of the 16 papers included seven studies conducted by researchers or with participants in the USA with the remaining studies being conducted with patients from Egypt, India, Italy, Jordan, Sweden, Netherlands, UAE, and UK. No Australian researchers or Autistic patients were represented in any of the studies eligible for inclusion in this review.

Of the 16 included papers, two studies from Sweden by Blomqvist et al. studied adult participants with a mean age of 33 years, interestingly these studies showed that the ASD group had a higher incidence of buccal gingival recession and hyposalivation associated with medication than the neurotypical control group but that the incidence of caries was similar for both [27,28]. In the majority of the studies, the participants were children and the gender distribution in the studies was representative of a male-to-female ratio of 4:1. The Autistic adults in this study tended to miss appointments due to forgetfulness and it was recommended that reminders were sent to address this [28]. Anxiety levels were higher for the Autistic adults and this correlated with reporting that 22% of those with ASD were being forced to have treatment compared with 2% of the neurotypical, control group [28]. Similarly pain during dental treatment, insufficient anesthesia as well as pain during other medical treatments were reported as higher for the ASD group [28]. These factors are shown to result in increased anxiety and were identified as barriers to accessing dental care for this group [28].

In the study from India by Dave et al. Autistic participants were divided into groups with age ranges from 5 to 20 years and 21 to 35 years [30]. A self-administered questionnaire was completed by 64 parents to determine their knowledge of oral health care and revealed that the majority of parents were not aware that oral health is related to general health [30]. Therefore, it was suggested that an oral health promotion program be introduced to raise awareness of the importance of oral health and thereby improve the oral health quality of life of Autistic persons [42]. 

In the qualitative study by Como et al., seven dental practitioners with experience treating Autistic children attended two focus groups [29]. This study identified that unconscious bias amongst the dental team has a negative impact on access to oral health care for Autistic persons and that further education is needed to address the language used and the attitude of clinicians. In 2022 Como et al. also assessed the “Oral care knowledge, attitudes, and practices of Black/African American caregivers of autistic children and non-autistic children” [43] and concluded that whilst caregivers demonstrated negative dental experiences they were aware of the need to access good oral health care for their Autistic children [43]. This was also observed by Floríndez et al. who recommended that families work with clinicians to develop effective oral health care, and therefore, reduce the burden of poor oral health in this disadvantaged population to reduce oral health inequities for Autistic individuals [8].

This scoping review endeavored to scope the evidence on the barriers to oral health care for Autistic individuals. Relevant findings were extracted into a data extraction form and were classified under five themes: difficulty in accessing suitable clinicians; negative past dental experience of Autistic individuals, parents, and careers; parental perceptions of the impact of ASD when accessing oral health care; clinician bias and clinician education. Thematic findings suggest the barriers reflect an interrelated combination of factors perceived by the recipients of care (parents, carers, and individuals) and the providers (dental practitioners). Difficulties in negotiating the communication, behavioral and sensory features of Autistic individuals have been identified by parents, carers, and dental practitioners treating Autistic individuals. To address the oral health care needs of Autistic patients, it is necessary to understand the current barriers to oral health care for these patients. In this scoping review, difficulty finding a dental practitioner able to treat Autistic individuals, negative past dental experience, and parental perceptions as well as clinician bias and education have been identified as barriers.

### 3.1. Difficulty in Accessing Suitable Clinicians

Finding a dental practitioner willing and competent to treat Autistic individuals has been proven to be a barrier to accessing dental care. Of the 16 papers, 8 papers reported that finding a dentist willing or competent to treat an Autistic individual was a major concern for parents and carers [23,27,28,31,32,34,36,40]. Kind et al. noted that that only 10% of American general dentists perform dental treatment for individuals with special needs including ASD, 21% of parents were dissatisfied with the dental care they received and 88% of parents could accurately predict a child’s cooperation for dental treatment [34]. Indeed, as described by Logrieco, parents of Autistic individuals reported experiencing a great deal of stress during a dental visit due to concerns over their own knowledge of preventative oral care and/or the preparedness of the dental practitioner to accommodate their child’s potential fears of the dental environment, communication difficulties, aggressive behaviors, difficulties expressing oral pain and/or sensory sensibilities [36]. Alshatrat et al. also highlighted parents’ identification of a lack of knowledge, lack of specialised staff and inadequate facilities to support the treatment of individuals with disabilities in their case-controlled study [23]. Similarly, Duker et al. conducted focus groups in their qualitative study which highlighted the difficulty of “finding the right dentist” as well as issues such as feeling uncomfortable with all the sensory stimulations associated with a traditional dental environment, the need for restraint and the negative impact this had on their Autistic male children [31]. Parents reported that when restrained, it appeared that their sons were being tortured and that their dental experience was very uncomfortable due to sensory sensitivities associated with ASD. Caregivers reported experiencing challenges in providing dental care such as toothbrushing both at home as well as those experienced in the traditional dental environment [31].

Furthermore, as identified in the DSM5, the criteria for the diagnosis of ASD and the behaviors of Autistic individuals, require special skills to facilitate the treatment depending on the severity of the symptoms [1]. Not all clinicians are prepared or trained to provide these accommodations competently and confidently to patients with special needs and so this is considered a barrier. Therefore, difficulty finding a suitably trained and experienced clinician, with the required compassion and understanding of the needs of Autistic patients, has been identified as a barrier to accessing and receiving dental treatment. Not having a regular dentist or not being able to find a dentist willing to treat an Autistic patient limited their access to appropriate treatment options [28]. Similarly, Taneja et al. reported that finding a dentist who was willing and able to adapt to the behavioral and oral health needs of their child was the major barrier to accessing dental care and achieving oral health [40]. Further education is needed for undergraduate and graduate dental practitioners to address the lack of availability of general dental practitioners who are suitably confident and competent to treat all patients of all abilities.

### 3.2. Negative Past Dental Experience of Autistic Individuals, Parents, and Careers

Previous negative dental experience and parents/carers’ failure to prioritize oral health were barriers identified in five of the included papers in this scoping review [27,30,31,32,34]. Dave et al. surveyed 64 Autistic individuals, aged between five to 35 years, and noted that 18% of parents and carers lacked awareness of the importance of oral hygiene and its impact on general health [30]. In a survey of Dutch Autistic children, Kind et al. also noted that children of parents who are themselves irregular attenders for dental treatment are also irregular attenders [34].

Further research is required to develop education for undergraduate and postgraduate general dental practitioners to provide a positive dental experience. Education needs to be aimed at developing the clinician’s confidence and competence in the delivery of preventive dental care for Autistic patients. This will result in a positive change to the experience of dental treatment and address their negative past experience as patients, parents and carers and reduce the burden of unmet dental care.

### 3.3. Parental Perceptions of the Impact of ASD When Accessing Oral Health Care

The previous Section 3.1 (Difficulty in accessing suitable clinicians) identified difficulty in accessing suitable clinicians for Autistic individuals and this theme is also addressed in this section as a parental issue. There is overlap in the themes identified due to the inter-related combination of factors perceived by the recipients of care (parents, carers, and individuals) and the providers (dental practitioners).

Parental perceptions were noted as a major concern in eight of the 16 papers in this scoping review [23,29,33,35,37,38,39,41]. Some parents and carers reported being overwhelmed and/or embarrassed regarding the behavior and lack of cooperation of the Autistic individual [23,35]. Parents also report that there is a lack of specialist dental staff, inadequate facilities in the dental environment, dentists are inadequately trained, waiting lists are long, difficulties associated with access to dental clinics and parking, financial issues and absence of insurance to cover dental costs [23].

Parents who are overwhelmed by their children’s special needs and behavioral problems are unable to focus on home care such as toothbrushing and have little energy left for oral care including regular dental visits [35]. For parents, the management of an Autistic child can result in fatalism and fear and so oral health is not always a priority in view of the overwhelming medical and behavioral issues that these families must manage [33].

In the study by Hammersmith et al., a multi-site randomised clinical trial was conducted and identified that 46% of caregivers for Autistic persons expect that the majority of children will develop dental cavities [33]. This paper identified an oral health fatalistic view and a higher incidence of unmet dental needs for Autistic persons [33]. Further research to address the inequities in oral health is recommended to reduce the higher incidence of dental disease amongst this population and to educate parents and carers on the importance of good oral health [33].

### 3.4. Clinician Bias

Families and their children with autism often feel stigmatised in a healthcare setting due to various factors [29]. Two of the 16 papers identified that clinician bias and attitude toward Autistic individuals were a major concern [29,41]. Como et al.’s qualitative study of the language used by dental practitioners to describe their experiences providing oral health care to Autistic individuals revealed implicit bias among these health care providers [29].

The three themes reflecting this implicit bias were the micro-aggressive attitude of the dental team, the marginalisation of individuals with special needs and preconceptions of the clinicians [29]. Hyper-empathy of the Autistic child and the parent’s inability to advocate for their child due to a perceived medical authority is also associated with a lack of willingness of the dental team to adapt to special needs [29]. Furthermore, this study identified a need for flexibility by the dental team so that the parents and carers feel confident in the treating clinician, and have a plan for the continuation of treatment and a clear referral pathway for specialist dental treatment, should the need arise [29].

The unconscious bias of the dental team negatively influences “patient-provider rapport” [29] and there needs to be further education to address cultural competency in the delivery of preventive oral health care by general dental practitioners to ensure equitable delivery of oral health care to the spectrum of needs and abilities of Autistic persons. It is important that dental practitioners and the dental team reflect on the language and communication used when treating Autistic patients so that they can understand their own unconscious biases and thereby deliver an equitable dental experience. Stereotyping by ability, race, or gender is not supported by evidence and clinician pre-conceptions and inappropriate use of negative language are evidence of unconscious bias and influence treatment outcomes [29]. Como et al. recommend that “it is crucial that Health Care Practitioner training include foundational preparation for treating diverse populations” [29]. Whilst the findings from this paper do not represent the opinions of all the participants in the focus groups they show the “subtle undertones in the language used by some providers” when referring to their patients and their parents and carers [29]. Research shows that “perceived, as well as overt, discrimination” in the language of dental practitioners results in a suboptimal mental and physical health outcome [29] and is, therefore, a barrier to oral health for Autistic persons.

Similarly, in the study by Thomas et al., parents of male and female children aged 4–13 were interviewed and four main themes emerged from the data analysis [41]. The results highlighted the need for flexibility in the dental environment; the need for parents to be able to advocate for their child’s needs; the availability of treatment beyond the examination and the provision of a clear referral pathway for specialist treatment if needed [41].

Barriers for Autistic individuals included hyper-empathy, the dental chair, concerns with the length of time spent in the waiting room, parents’ perception of the value of effective communication and the willingness of the dental team to listen and adapt to a child’s individual needs [41].

### 3.5. Clinician Education

Dental Practitioners reported that a lack of undergraduate and postgraduate education and training was a barrier to care in seven of the included papers [23,29,32,35,39,40,41]. American dentists acknowledge that they have received minimal education in the care of children with special needs and recognise that there is a need for dentists to be provided with training in the management of Autistic individuals [35]. General dentists are often required to treat Autistic individuals, but they can lack the training and experience to provide appropriate oral health care including management of sometimes-challenging behavior [35]. There are a limited number of providers with extensive experience with ASD; dental practitioners need to be calm and understanding, regardless of the behavior of the individual [7,39].

All barriers identified in this scoping review indicate that further education of undergraduate Dental Practitioners is needed to adapt to the individual needs of the Autistic person. The dental environment can be difficult for Autistic persons due to sensory and behavioral challenges and the reluctance of dental practitioners to treat them and there is extensive evidence to support the need to examine the sensory and behavioral challenges of autistic individuals [8,9,41,43,44,45]. In a UK study, Thomas et al. conducted interviews with parents of Autistic children aged 4–13 years and from the collected data the main themes identified were a lack of flexibility by the dental team and the environment; lack of confidence in the parents to advocate for their child’s individual needs and a lack of treatment following an examination with unclear referral pathways to specialist dental care [41]. Parents also reported that there is a need for the dental environment to be adapted with consideration of the sensory, emotional, and physical needs of Autistic persons and this necessitates education of the entire dental team, not only the treating clinician. Adapting the environment to the individual needs of a person may include adapting the physical appearance, verbal and non-verbal communication of the dental team and ensuring there is sufficient time to explain procedures to the child and parent in a friendly, sensory-adapted environment [41]. By educating the entire dental team and not just the treating dental practitioner a better oral health outcome could be achieved thereby this barrier could be addressed. Developing a strong relationship between the patient, parent, carer and the dental team [41] will enable a consistent, standardised approach to treatment and thereby reduce the burden of disease and unmet dental needs of this population.

## 4. Discussion

Findings from this scoping review highlight the need for change to address the barriers experienced by Autistic individuals and their families from seeking oral health care or managing their own oral health. The implications of this study support the need for dental practitioners to be educated at an undergraduate and postgraduate level to enable them to confidently treat Autistic individuals and thereby provide equitable access to oral health care. Notably, not all dental practitioners will choose to provide oral health care to individuals with special needs and may restrict their practice accordingly. However, primary prevention and treatment can be provided by the dental team with appropriate training on the needs of Autistic individuals, thereby reducing the burden of dental diseases.

Providers have to adapt to the individual needs of all patients when delivering oral health care for Autistic people. Identifying and understanding the barriers that are presented to Autistic children and adults and developing ways to deliver treatment in a sensory-adapted environment free of bias and sensitive to the patients’ needs is needed to ensure that their dental needs are met and there is equitable access to oral health care.

Nelson et al. utilised educational philosophy to provide a more understanding approach to the care of ASD patients. Preparation for the dental visit and discussion with the parents or caregivers allowed the clinician and parent or caregiver the opportunity to assess issues and make accommodations, to facilitate a positive first appointment experience for the patient. Nelson et al. recommended that at the first visit, the patient simply observed and experienced the treatment environment to allow them to “participate without anxiety brought on by the unknown” [20]. It was recommended that appointments should be short, and the clinician needs to ensure the patient is seen at the appointed time [20].

Adapting to the individualised needs of Autistic patients can include electronic communication systems that prepare the patients for their experience at a dental clinic and address their fear of the dental environment [46,47]; adapting the environment to the sensory needs of Autistic individuals to reduce physical and behavioral stress [7,11]; familiarisation by using visual model techniques [48]: as well as pre-treatment questionnaires and social stories to enable the Autistic patient and the dental practitioner prepare for their dental visit [49].

Further research is needed to define the treatment currently provided by dental practitioners who treat Autistic individuals, evaluate how they negotiate the behavioral challenges of these individuals and define the skill set of those dental practitioners who choose to treat Autistic individuals. The goal of this further research would be to develop guidelines for a model of care for individuals with ASD and associated educational curriculum.

Various limitations were identified in the included papers. Most studies related to the provision of care for children could limit the generalizability of the findings to adults with ASD and/or dentists who work with such individuals. Most of the included studies were conducted from the perspective of the parents, carers and/or dental practitioners rather than the Autistic individuals. Further co-designed research is needed to fully understand the barriers as perceived by individuals with ASD to facilitate the development of further education for Dental Practitioners, persons with ASD and their Parents and Carers. Future research should include the voices of those with lived experience of ASD so that further education for undergraduate and graduate dental practitioners incorporates the required sensory, emotional, and physical adaptations to the dental environment and addresses the unconscious bias of the dental team to provide an equitable positive dental experience for persons with ASD.

## 5. Conclusions

Autistic individuals are known to experience poorer oral health outcomes when compared to their neurotypical counterparts. This scoping review sought to investigate the barriers to oral health for people living with ASD. The findings in this review support the need for the development, testing, and implementation of appropriate and achievable autism-focused interventions and their inclusion in the development of an educational curriculum for dental practitioners from the undergraduate to postgraduate levels to improve the provision of equitable oral health care to Autistic individuals.

## Figures and Tables

**Table 1:** Inclusion and exclusion criteria.

Included	Excluded
Written in English	Excluded as not English
Full text available	Full text unavailable
Related to topic—Barriers, ASD, Oral Health	Excluded as not barriers to oral health care for Autistic patients
Adults and middle adolescents (≥15 years) and Children (<14 years)	Include all ages, no exclusion regarding age of population studies
Dates searched 2014–current	Excluded if written before publication of DSM-5 in 2014
Case Controlled Studies, Clinical Study, Qualitative Descriptive Studies, Random Controlled Trials	Excluded case reports, case series, reviews including systematic reviews, and opinion pieces

**Table 2:** Data Extracted with specific themes identified within the included studies.

Author/Year/ Country of Origin	Research Design	Target Population	Research Method	Major Themes/Barriers Identified	Barriers Identified
Alshatrat et al., 2020/ Jordon [23]	Case Control Study	Parents/carers of Autistic Individuals (n = 147) compared to without ASD (n = 149)	Questionnaire	Access Parental Perceptions Education	Embarrassment Lack specialist staff Uncooperative behavior. Facilities/environment Long waiting lists Dentist too far away No parking Financial. Finding a dentist willing and able Dentist education
Blomqvist et al., 2014/ Sweden [27]	Clinical Study	Autistic group (n = 47) and non-autistic group (n = 69)	Questionnaire	Negative past experience Access	Reduced salivary flow due to stress and anxiety increases oral health risks. Missed appointments as forgot or unable to attend due to illness, work, school, financial. Finding a dentist
Blomqvist et al., 2015/ Sweden [28]	Cross sectional study	ASD group (n = 47) and non-ASD group (n = 69)	Questionnaire and dental examination	Access	Stress and anxiety Negative dental experience Pain during dental Inadequate LA Sensory over responsiveness. Communication Feeling helpless in dental chair, forced to have treatment and not adequately prepared.
Como et al., 2020/ USA [29]	Qualitative	dental providers n = 7	Focus Groups	Parental Perceptions of the impact of ASD Bias Education	Stigma and marginalisation Unconscious bias of the treating clinician and dental team
Dave et al., 2020/ India [30]	Cross-sectional study	Parents of autistic patients (n = 64)	Questionnaire	Negative experience	Lack of caregiver awareness of significance of oral hygiene and impact on oral health Poor knowledge, attitude, and practice amongst parents of Autistic children Need for oral health promotion in special care for children and parents
Duker et al., 2017/ USA [31]	Qualitative study	Parents of male autistic children 5–18 years (n = 13)	Focus Groups	Negative experience Access	Difficult to find dentist. Sensory sensitivities Restraint Drugs Caregiver experiences at home and at dentist
El Khatib et al., 2014/ Egypt [32]	Case control study	Parents of autistic children (n = 100) and non-autistic children (n = 100)	Questionnaire and Dental Examination	Negative experience Access Education	Behavior and life factors Limited access to dental care Dental Education Oral health low priority
Hammersmith et al., 2021/ USA [33]	RCT	Multi-site 6-month RCT of parent-training intervention. Families of Autistic children aged 3–13 years. (n = 118)	RCT	Parental Perceptions	Fatalistic belief of parents Cultural beliefs Costs, family stress External influences Access to dental care and commercial products
Kind et al., 2020/ The Netherlands [34]	Cross sectional study	Parents of autistic children (2–18 years) questionnaires returned (n = 246)	Questionnaire	Negative experience Access	Dissatisfaction with dental care Lack of cooperation for dental treatment Lack of general dentists to perform dental treatment for children with special needs. Parents are not regular dentist attenders. Unmet dental needs
Lewis et al., 2015/ USA [35]	Cross sectional study	Focus groups of parents of autistic children aged 3–17 years old (n = 4)	Focus Groups	Parental Perceptions Education	Fear and anxiety, behavior. Parents overwhelmed. Low priority for oral health Waiting times Dentists’ lack of education in the care of Autistic children
Logrieco et al., 2020/ Italy [36]	Cross-sectional study	Parents of typical development children (n = 275) 57 parents of autistic children 3–15 years old (n = 57) dentists (n = 61)	Questionnaires	Access	Parents’ fatalism, Caregivers’ demographic Scarce presence of experts Parents fear Finding a dentist Communication and social interaction Behavior Higher sensory sensitivities Bright lights and tastes Medications and xerostomia Financial
Mansoor et al., 2018/ UAE [37]	Case-controlled comparative study	Autistic Children (n = 84) Healthy children attending special needs centers and schools in Dubai including siblings of the autistic children (n = 53)	Questionnaires of parents and guardians	Parental Perceptions	Uncooperative patient Need for physical restraint. Negative experiences at dentist due to behavior Finding a dentist.
Stein et al., 2014/ USA [38]	Cross-sectional study	Measurement of behavioral and psychological distress in children aged 6–12 receiving routine dental treatment. (n = 44)	Cross-sectional study	Parental Perceptions	Stress Uncooperative Need for restraint.
Stein et al., 2019/ USA [39]	Qualitative study	Groups of Parents of Autistic children (n = 2) Groups of Dentists treating Autistic Children (n = 2)	Focus groups	Parental Perceptions Education	Limited number of providers with extensive experience with ASD
Taneja et al., 2020/ USA [40]	Cross-sectional study	Caregivers of an Autistic child (n = 46) Non-autistic children with chronic health issues (n = 37)	Surveys	Access Education	Difficulty finding a dentist who would treat their child Child’s uncooperative behavior Fear Lack transportation Time constraints
Thomas et al., 2018/ UK [41]	Cross sectional study	17 parents of children with a diagnosis or working diagnosis of autism	Semi-structured interviews	Parental Perceptions Bias Education	Hyper-empathy The dental chair Time in waiting room. Parents ability to advocate for their child Parents perception of value of effective communication Willingness of the dental team to listen and adapt to a child’s individual needs Dentist lack of training Lack of flexibility Lack of confidence of parent Lack of continuation of service, clear referral pathway to specialist dental services
